# Diagnostic Accuracy of Ultrasound B scan using 10 MHz linear probe in ocular trauma;results from a high burden country

**DOI:** 10.12669/pjms.322.9241

**Published:** 2016

**Authors:** Muhammad Kashif Shazlee, Muhammad Ali, Muhammad SaadAhmed, Ammad Hussain, Kamran Hameed, Irfan Amjad Lutfi, Muhammad Tahir Khan

**Affiliations:** 1 Muhammad KashifShazlee, FCPS. Department of Radiology, Ziauddin University Hospital, Karachi, Pakistan; 2 Muhammad Ali, FCPS. Department of Radiology, Ziauddin University Hospital, Karachi, Pakistan; 3 Muhammad Saad Ahmed, FCPS. Department of Radiology, Ziauddin University Hospital, Karachi, Pakistan; 4 Ammad Hussain, FRCR. Department of Radiology, Ziauddin University Hospital, Karachi, Pakistan; 5 Kamran Hameed, FCPS. Department of Radiology, Ziauddin University Hospital, Karachi, Pakistan; 6 Irfan Amjad Lutfi, MD. Department of Radiology, Ziauddin University Hospital, Karachi, Pakistan; 7 Muhammad Tahir Khan, M.Phil. School of Public Health, Dow University of Health Sciences, Karachi, Pakistan

**Keywords:** Ultrasound, Ocular Trauma, Diagnostic accuracy

## Abstract

**Objective:**

To study the diagnostic accuracy of Ultrasound B scan using 10 MHz linear probe in ocular trauma.

**Methods:**

A total of 61 patients with 63 ocular injuries were assessed during July 2013 to January 2014. All patients were referred to the department of Radiology from Emergency Room since adequate clinical assessment of the fundus was impossible because of the presence of opaque ocular media. Based on radiological diagnosis, the patients were provided treatment (surgical or medical). Clinical diagnosis was confirmed during surgical procedures or clinical follow-up.

**Results:**

A total of 63 ocular injuries were examined in 61 patients. The overall sensitivity was 91.5%, Specificity was 98.87%, Positive predictive value was 87.62 and Negative predictive value was 99%.

**Conclusion:**

Ultrasound B-scan is a sensitive, non invasive and rapid way of assessing intraocular damage caused by blunt or penetrating eye injuries.

## INTRODUCTION

Estimates on global data suggest that each year around 1.6 million people become blind due to ocular trauma, while an additional 2.3 and 19 million suffer from bilateral low vision and unlilateral blindness/low vision respectively.^1^ Early diagnosis and intervention is of utmost importance in preventing blindness in such cases. In developing countries like Pakistan, the burden on healthcare system due to ocular trauma is overwhelming with around 7% admission in Ophthalmology ward related to ocular trauma.^2^ Such injuries can readily be investigated by ultrasound, which is of particular value when the light conducting media are opacified by hemorrhage or other injury.^3^

Ultrasound B-scan (brightness modulation) gives exceptionally detailed bi-dimensional images of cornea, anterior sclera, aqueous chamber, anterior chamber angle structures, lens and vitreous etc.^4^-^7^ Before surgery of the traumatized eye it is helpful to have knowledge of the degree of internal derangements; B-scan has proved the ideal tool that provides information about vitreous hemorrhage, lens dislocation and rupture, detachments of the coats, globe rupture and foreign bodies etc. severe complication are treated by microsurgical techniques, and ultrasound evaluation represents the only practicable method of examination for surgical planning.^8^

Various studies have reported the diagnostic accuracy of Ultrasound in the detection of different forms of ocular trauma in different populations. Vrablik ME and associates reported the sensitivity and specificity of ultrasonography in Retinal Detachment to be ranging between 97%-100% and 83%-100% respectively.^4^ Furthermore, for lens dislocation, SHO Haghighi et al.^1^ reported the sensitivity and specificity of ultrasonography were 84.6% (95% Cl: 53.7-97.3) and 98.3% (95% Cl: 93.3- 99.7), respectively.^5^

Pakistan has a very high burden of injuries which include unintentional and intentional injuries. The overall prevalence of unintentional injuries in 2007 was around 46 per 1000 per year.^9^ However, in recent years, terrorism related blast injuries have also significantly increased the burden on emergency rooms in the country.^10^ Karachi, the biggest city of the country with a population of 20 million observes high burden of injuries coming from road traffic accidents, domestic trauma and terrorist attacks in the form of bomb blasts.^11^ All these pose a great burden on the health care system and a very few tertiary care institutes exposes the capability to cater such patients. In this background. Our objective was how effective and diagnostically valid ultrasound B scan is, which is an operator dependent technique with a generally high load of patients turnover.

## METHODS

A total of 61 patients with 63 ocular injuries were assessed during July 2013 to January 2014. All patients were referred to the department of Radiology from Emergency Room since adequate clinical assessment of the fundus was impossible because of the presence of opaque ocular media. Ultrasound were performed with 10 MHz transducer (Toshiba Diagnostic ultrasound equipment with probe selector unit. Model no. SSA-304A). Patients, who were in a supine position on the examining couch, underwent Ultrasound with the transducer placed gentle on the closed eyelid. Through-the- lid contact imaging was employed with a standard water-soluble coupling gel, using a very gentle technique to cause minimal discomfort to the injured eye. Children tolerated the examination well. The scan was conducted by single radiologist for all 63 eyes. Based on radiological diagnosis, the patients were provided treatment (surgical or medical). Clinical diagnosis was confirmed during surgical procedures or clinical follow-up.

The data was entered and analyzed using Statistical Package for Social Sciences (IBM SPSS v20 for Windows, Chicago Illinois). Frequencies and percentages were calculated for categorical data (gender, diagnosis) while mean and standard deviation was calculated for total procedure duration (from scanning till reporting) while mean and 95% confidence interval was calculated. Kappa statistic was calculated for each condition to the agreement beyond chance between radiological diagnosis and surgical diagnosis.

## RESULTS

A total of 63 ocular injuries were examined in 61 patients (43 males and 18 females) with mean age of 53 years (95% CI 41-65). The causes of ocular injuries included 56 (89%) due to road traffic accidents, 4(6%) due to outdoor violence, 2 (3%) due to accidental falls, and 1(2) domestic.

The mean procedure duration from scanning till reporting was 28.7 minutes (± 12 SD). Radiological diagnoses based on ultrasound B scan were as follows. Vitreous Hemorrhage 23, Post traumatic cataract 16, Retinal detachment 08, Lens dislocation 05, Choroidal detachment 03, Foreign bodies 05, Partial retinal detachment 02 and posterior vitreous detachment in 01 patient. The parameters of diagnostic accuracy when compared with surgical and/medical procedures are shown in Table-I.

**Table-I:** Diagnostic accuracy parameters of grey scale ultrasound B scan for different conditions resulting from oppacifying ocular trauma (n=63).

Condition*	Diagnosis		Sensitivity (95%CI)	Specificity (95%CI)	PPV (95%CI)	NPV (95%CI)	Validity (95%CI)	Kappa (p-value)

	Surgical	Clinical						
Vitreous Hemorrhage (n=24)	14	10	96 (79.8-99.3)	100 (91.0-100)	100 (82.1-100)	98 (85.2-99.8)	98 (95.3-100))	0.96 (<0.001)
Post Traumatic Cataract (n=15)	10	5	100 (79.6-100)	98 (89.1-99.6)	94 (67.7-99.6)	100 (90.1-100)	98 (95.3-100)	0.95 (<0.001)
Retinal Detachment (n=09)	7	2	89 (56.5-98.0)	100 (93.4-100)	100 (59.7-100)	98 (89.0-99.9)	98 (95.3-100)	0.93 (<0.001)
Lens Dislocation (n=05)	5	0	80 (37.5-96.4)	98 (91.0-99.7)	80 (29.8-98.9)	98 (89.5-99.9)	97 (92.5-100)	0.78 (<0.001)
Choroidal Detachment (n=03)	3	0	100 (44.0-100)	100 (94.0-100)	100 (31.1-100)	100 (92.5-100)	100 (100-100)	1 (<0.001)
Foreign Bodies (n=03)	2	1	100 (43.8-100)	97 (88.6-99.0)	60 (17.0-82.9)	100 (92.2-100)	97 (92.5-100)	0.73 (<0.001)
Partial Retinal Detachment (n=03)	3	0	67 (20.7-93.8)	98 (91.1-99.7)	67 (12.5-98.2)	98 (89.8-99.9)	97 (92.5-100)	0.65 (<0.001)
Posterior Vitreous Detachment (n=01)	1	0	100 (20.6-100)	100 (93.9-100)	100 (54.6-100)	100 (92.7-100)	100 (100-100)	1 (<0.001)

* Final diagnosis was mad e through surgical procedures and/or clinical follow-up.

PPV: Positive predictive value, NPV: Negative predictive value.

## DISCUSSION

Ocular trauma can render ocular media opaque due to hyphema or hemorrhage causing evaluation of the posterior segment difficult or impossible. Traumatic cataract may develop within hours and prevent visualization of the fundus. In such a state early diagnosis with accuracy is hindered due to difficulty in examination of the eye. However, ultrasound B scan has proven to be a precise and valid alternative aiding in the prompt and early diagnosis in cases of ocular trauma where opacification of the light conducting media has occurred. This is especially important in a setting where the overall burden of trauma on healthcare in general and on emergency department in particular is increasing every year. This study which was done in Karachi city, which houses over 20 million people has been adversely affected by intentional and unintentional injuries ranging from road traffic accidents to domestic violence and from accidental injuries to injuries caused by terrorism attacks and bomb blasts.^9^,^12^

The findings of our study report an overall 98% diagnostic validity of ultrasound B scan in detecting ocular trauma. In five out of eight conditions ultrasound showed excellent agreement with surgical diagnosis while very good agreement in the rest three. Although ultrasound based diagnosis is very much operator dependant and the accuracy may vary between settings, other studies from different regions have also reported similar results. Kim S et al.^13^ found the ultrasound to be sensitive in 73%, specific in 90%, and Imran S et al.^14^ reported ultrasound to have 84.6% sensitivity and 96.5% specificity for detecting vitreous haemorrhage.

Our study demonstrated 100% sensitivity of ultrasound B scan, while 97 specificity in diagnosing foreign body due to ocular trauma, however Deramo et al.^15^ showed that ultrasound is 75% sensitive in detecting ocular foreign bodies, that is contrast to physical examination of the eyes which even failed to detect majority of the cases. Another study by Amanullah et al revealed 93% sensitivity of ultrasonography (US) for all kind of intraocular foreign bodies including metallic and non-metallic.^16^ The main impediment in clinical detection of foreign body in a traumatized eye is small size, limited patient’s compliance, optical media haziness and concealed location. Hence the US in an effective mode of excluding other diagnosis and format a plan of further intervention.

In the emergency department Ultrasound proves to be more cost effective and a better screening tool for patients presenting with ocular trauma, specifically in low income countries like Pakistan. An excellent agreement between Ultra Sound findings and clinical diagnosis beyond chance is also in concurrence with other studies reported from different parts of the world.^17^,^18^ The findings of this study are comparable to those of two other large series assessing ocular injuries by US B-scan. Both studies showed diagnostic accuracy of greater than 90%. Furthermore, in our study the diagnostic validity was found to be comparable with that of CT scan as reported by Imran S et al.^14^

The mean evaluation time taken from the presentation of the patients to diagnosis for ultrasound B scan in ocular trauma is considerable low and the portability of the device makes it very practical to be used in emergency room setups. Our study reported a considerably low mean evaluation time for ocular trauma despite high turnover of patients in our setup. Delay in diagnosis in traumatic eye injuries have been recognized as a major cause of preventable visual impairment.^19^

Real-time ultrasound B-scan is an essential addition to clinical evaluation of the traumatized eye with opaque media. To our knowledge, it is the only-non-invasive mean of assessment of the posterior segment in these cases. It allows the assessment of the presence and extent of primary vitreous haemorrhage and the possible early diagnosis of retinal detachment. In cases in which surgery is not considered, ultrasound is useful as a follow-up examination to assess resolution (clearing of vitreous haemorrhage without fibro vascular band formation) and to exclude development of retinal detachment. Ultrasound B-scan is recommended as an initial diagnosis tool and should be made a routine practice in ocular injuries especially those involving posterior segment.

## CONCLUSION

Ultrasound B-scan is an excellent, non-invasive, rapid diagnostic tool in assessing intraocular damage caused by blunt or penetrating eye injuries which most of the time renders ophthalmoscopy impossible due to opacification of light transmitting media. The images provide essential and detailed information about soft tissues damage, helping in the decision regarding early surgery, before chronic changes have occurred.
